# Survey on Reconnaissance Autonomous Robotic Systems for Disaster Management

**DOI:** 10.3390/s26051659

**Published:** 2026-03-05

**Authors:** Sahaj Sinha, Sinjae Lee, Saurabh Singh

**Affiliations:** 1 AI and Big Data, Endicott College, Woosong University, Daejeon 34606, Republic of Korea; 2 Department of Computer and Software Major, School of IT Convergence, Woosong University, Daejeon 34606, Republic of Korea; sinjaelee@wsu.ac.kr

**Keywords:** autonomous robotics, disaster management, computer vision, machine learning, UGV, ground reconnaissance systems, Arduino control, LoRa communication

## Abstract

Systems that operate in dangerous environments are becoming essential in case of emergencies. This survey reviews the latest ground reconnaissance robots using computer vision (CV), machine learning (ML), MCU-based control, LoRa communication, DC motors, and dual-power systems. The analysis includes hardware and algorithms, and their performance in the field and lab. There has been clear progress in navigation, sensor fusion, and situational awareness. The main challenges which remain include the use of energy and standardization of benchmarks. This survey focuses exclusively on Unmanned Ground Vehicles (UGVs) for disaster reconnaissance, examining recent advances in hardware, software, and autonomy. The survey highlights the improvements in navigation, sensor fusion, and intelligence, and identifies remaining challenges such as energy limitations, robustness in harsh conditions, and the lack of standardized benchmarks. The analysis synthesizes findings from over 190 recent studies (2020–2025) in ground-based disaster robotics, providing a comprehensive overview of current capabilities and research gaps. It encapsulates all issues with their remedy for future disaster-response systems.

## 1. Introduction and Background

The increasing frequency and severity of natural disasters globally have led to a growing imperative for advanced emergency response systems that can operate in environments too hazardous for human personnel [1,2,3]. Autonomous ground robots have become critical tools in disaster-response, undertaking tasks too dangerous or impractical for humans. These UGVs provide vital situational awareness and enable safer operations in collapsed buildings, toxic environments, and hazardous terrain [4,5,6,7].

**Figure 1:**
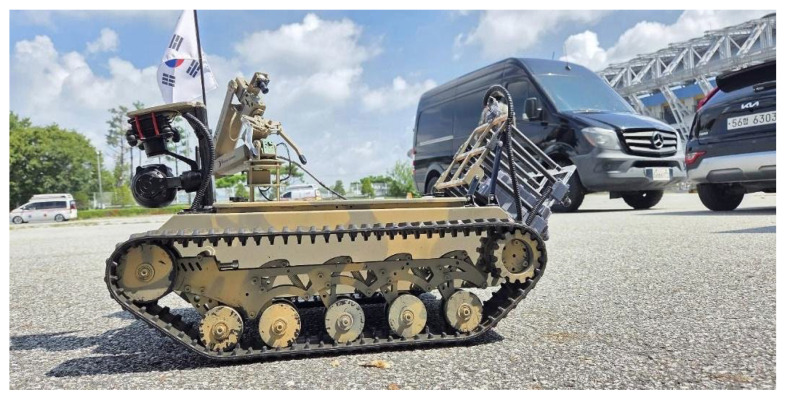
Representative unmanned ground vehicle (UGV) platform for disaster reconnaissance, illustrating the chassis structure, sensor placement, and mechanical configuration. The figure is provided for conceptual visualization only and does not report experimental results.

UGVs have been deployed in real disasters, showcasing their utility in critical search and assessment tasks. For instance, these rovers were used in the rubble at the World Trade Center after the September 11 attacks to search for survivors [8]. Early use cases demonstrated the promise of ground robots, such as small robots carried into the WTC rubble in 2001 and larger ground vehicles used to inspect levees after Hurricane Katrina [9]. The utilization of UGVs was again observed during the 2005 Hurricane Katrina response and the 2011 Fukushima disaster-response. While these initial deployments provided enhanced situational awareness and reduced risk to human responders, they concurrently exposed inherent limitations in autonomy and ruggedness. These early deployments highlight why current research emphasizes full autonomy, ruggedness, and multi-modal sensing to overcome the limitations of earlier tethered and teleoperated systems. [10]. A representative UGV configuration used for disaster reconnaissance is illustrated in Figure 1.

This figure shows the real ground robot used for reconnaissance operations. It highlights the chassis, wheels, sensors, and overall mechanical design. The image helps readers understand the physical scale and configuration of the rover. It serves as the reference platform for all later system descriptions across various studies [11].

Learning from those experiences, modern disaster robotics has shifted toward fully autonomous rovers with advanced sensing and artificial intelligence capabilities [12]. Ground robots today can carry multiple sensors (cameras, LiDAR, gas detectors, etc.). They process data onboard via microcontroller units and operate for extended periods (typically 60–120 min) using efficient power management. Nevertheless, significant challenges remain; most notably, batteries constrain mission duration, harsh conditions (dust, smoke, complex terrain) can confound sensors. The lack of common evaluation metrics also makes it difficult to compare systems.

There is an immediate requirement for advanced autonomous ground vehicles that feature enhanced payload capacities for more sensors, advanced navigation capabilities, and onboard decision-making systems [13]. Current emergency scenarios strategies utilize autonomous ground robots to support human expertise, recognizing that the best results arise from collaborative operations in dangerous environments. This helps to leverage the specific strengths of robotic platforms [14]. The emphasis on robust deployment is amplified by economic assessments, which demonstrate that the early deployment of autonomous ground reconnaissance systems can reduce disaster-response costs by 25–40% through improved situational awareness and resource allocation. This economic justification suggests that UGV development is driven by substantial fiscal benefits alongside safety mandates, expanding the stakeholder base to include policy makers and recovery analysts [15]. The target audience of this survey includes robotics researchers, emergency response experts, and policy makers concerned with deploying autonomous rovers [16].

### 1.1. Technological Context and Evolution

Ground robots in crisis settings have evolved significantly alongside improvements in sensing, computation, and autonomy. Earlier generations of robots relied heavily on tethers or remote teleoperation, which provided limited environmental understanding [17]. Modern platforms benefit from advances in sensor technology. Some of them are compact LiDAR, thermal cameras, and Inertial Measurement Units (IMUs), coupled with machine learning techniques, enabling them to build 3D maps and detect hazards autonomously [18].

Innovations in communication, specifically low-power long-range radio technologies like LoRa, and decentralized algorithms allow teams of ground robots to coordinate effectively over long distances [19]. Recent fielded systems integrate multipurpose hardware, including robust chassis and suspension systems for traversing rubble, dual-battery setups for extended endurance, and modular mounts for flexible payloads. The microcontroller, often MCU-based, provides a reliable real-time control core with separate power for computing and locomotion [20]. These mechanical and electronic improvements have elevated the technology readiness of core components (power systems, motor control, basic autonomy) to high levels. But advanced features such as multi-robot cooperation and fully autonomous navigation in cluttered environments are still maturing [21]. Current reconnaissance robots utilize computer vision (CV) and LoRa modules to perceive their surroundings and execute autonomous decisions driven by machine learning (ML) [22].

Disaster environments pose multiple combined challenges, including the absence of GPS, weak communication links, brief mission time constraints, limited onboard processing capabilities, and weather uncertainties [23]. The adoption of LoRa communication and low-latency video systems is specifically intended to maintain persistent connectivity for ground robots during these operations [24].

Recent studies have focused on reliable operation in GPS-denied disaster environments. LiDAR-based SLAM, which fuses sensor data with IMU measurements, has become a widely adopted method for consistent localization inside structures and collapsed buildings. Many different examples have demonstrated submeter drift over difficult, longer missions. The integration of visual–inertial odometry and thermal–visual fusion will give complementary localization and perception mechanisms when LiDAR odometry is impacted due to the dusty and smoky environment. Low-power, long-range technologies like LoRa and adaptive mesh networking are employed to manage communication uncertainty for traversing and telemetry links from ground robots to operator, through partial soils and debris. The use of light-weight deep learning models and edge AI accelerators to address the problem of limited onboard computation is gaining traction for enabling real-time perception with a reduced energy footprint and latency. It is clear from the above that localization loss, communication instability, environmental clutter, and computation limit have been the focus of such research in actual disaster situations.

### 1.2. Disaster-Response Requirements

Disaster scenarios impose unique and demanding requirements on UGVs. Platforms must be capable of navigating uneven debris, tolerating harsh conditions such as dust and heat, and maintaining reliable communications in environments where GPS or cellular signals are denied [25].

Consequently, these vehicle designs emphasize sensor fusion, which involves combining data from cameras, LiDAR, IMUs, and other sensors to robustly perceive surroundings and accurately detect survivors or hazards [26]. A crucial operational requirement is the ability to detect humans through visual or thermal signatures with high accuracy. This is often reported in the 85–92% range when using advanced deep Convolutional Neural Networks (CNNs) like YOLO variants [27].

Furthermore, ground robots must integrate seamlessly into emergency operations, requiring them to provide live video and telemetry to command stations, operate via intuitive interface protocols, and be deployable with minimal expert oversight [28]. Ground vehicles leverage their inherently higher payload capacity compared to aerial systems to carry specialized sensors, such as gas detectors, radiation probes, and acoustic arrays, as well as tools like manipulators or sample collectors. All of which aid situational assessment [29]. The overall architecture of modern UGVs balances mobility, endurance, and intelligence, targeting extended missions (up to 2 h) with rich sensor suites while executing efficient onboard AI for autonomy. The short operational duration (60–120 min typical) establishes energy management as a constant and critical technical constraint that permeates the design and performance analysis of these systems [30].

At present, redundancy-aware autonomy and multi-modal sensor fusion schemes are being integrated into these rovers to fulfill use case operational needs. RGB cameras are commonly fused with thermal cameras to spot survivors in the dark or in smoky conditions. In a similar way, navigation on rubble can take place with LiDAR IMU fusion when they do not see anything. Other works consist of energy-aware planning and adaptive motion control to maximize coverage under limited mission time.

Communications which are critical further capitalize on architectures that are of hybrid fusion, where LoRa is deployed for long-range TSA telemetry, while low-latency links support critical control messages. In the end, adopted engineered short deep neural networks onboard embedded processors are the enablers of autonomously making decisions without the need for any external infrastructure. Overall, we find that existing research is effective at handling major challenges in crisis settings deployment: degraded perception of the environment, intermittent communications, limited power availability, and limited onboard computational resources.

### 1.3. Survey Scope and Methodology

This survey provides a systematic review of research published primarily from 2020 to 2025, focusing on autonomous systems designed for disaster reconnaissance [31]. The analysis concentrates on fully or semi-autonomous ground platforms utilized for situational awareness in hazardous environments, which include urban collapse zones, fire zones, and flood-damaged areas.

The core literature corpus comprises 190 peer-reviewed papers sourced from major academic repositories, including IEEE Xplore, Springer, and arXiv [32]. Critically, the survey intentionally excludes aerial (UAV) systems and related studies, except in cases where they offer direct comparative performance data relevant to ground robot operations [33]. Key inclusion criteria for the selected papers were defined as follows: (1) an emphasis on ground-based robots or co-robots operating autonomously; (2) the utilization of sensors such as cameras, LiDAR, or IMUs; (3) relevance to core disaster-related tasks, including search-and-rescue, mapping, and hazard detection. Papers were categorized based on research type (e.g., simulation, experiment, algorithm development), and a detailed analytical review of their findings was performed [34].

To enhance reproducibility, a structured literature selection procedure was followed. Relevant publications were retrieved from major scientific databases. These include IEEE Xplore, Scopus, Web of Science, and Google Scholar, using keyword combinations such as “UGV disaster response,” “ground robot search and rescue,” “autonomous navigation in hazardous environments,” and “multi-robot coordination.” The initial search returned a broad collection of studies, which were subsequently refined through a multi-stage screening process. Duplicate records were removed, and remaining papers were first filtered based on title and abstract relevance. Articles focusing exclusively on aerial platforms or unrelated application domains were excluded unless their methodologies were directly transferable to ground robotic systems.

The shortlisted papers were then subjected to full-text review, and only peer-reviewed journal articles and high-quality conference publications addressing rover autonomy, perception, navigation, coordination, or deployment in disaster environments were retained. Each selected study was categorized according to research focus, including algorithm development, experimental validation, simulation-based analysis, hardware platforms, and comparative evaluation. This systematic screening and classification process resulted in a final dataset of 190 papers, which forms the basis for the qualitative and quantitative analyses presented in this survey.

## 2. Literature Review and Classification

This section presents a structured review of existing research on autonomous field inspection robots for disaster management, with the objective of identifying dominant research directions, validation practices, and technological gaps. The surveyed literature is systematically analyzed and organized based on research focus, methodological approach, and level of real-world validation. By classifying prior studies into categories such as algorithm development, simulation-based research, experimental validation, hardware platform design, and field deployment, this review provides a clear overview of how the domain has evolved over time. The classification framework enables quantitative comparison of research trends and highlights the imbalance between theoretical developments and practical deployments, thereby establishing a foundation for deeper analysis in the subsequent subsections.

Although this survey primarily focuses on UGVs, a limited number of UAV-oriented studies are referenced where their core methodologies are directly transferable to ground robotic platforms. These include perception pipelines, SLAM frameworks, multi-robot coordination strategies, and autonomy architectures that are largely platform-agnostic. Such works are cited to provide broader methodological context, particularly in areas where mature benchmarking and algorithmic development have been more extensively explored in aerial robotics. UAV-focused papers that did not offer transferable insights relevant to navigation, perception, coordination, or disaster-response autonomy were excluded from the final analysis. This selective inclusion ensures that all retained references contribute meaningfully to the design and evaluation of ground-based robotic systems.

In addition to application-specific studies, several recent surveys and benchmarking works have been incorporated to provide a broader perspective on autonomous navigation, perception, and multi-robot coordination in unstructured environments [35,36,37]. These studies offer comparative evaluations of algorithms and platforms, helping contextualize performance trends and technological maturity across different deployment scenarios. Their inclusion strengthens the background by situating UGV disaster-response research within the wider landscape of field robotics.

### 2.1. Search Strategy

A structured literature search was executed across major databases, including IEEE Xplore, SpringerLink, ScienceDirect, and arXiv [38]. Keywords used for the search included “ground robot”, “disaster”, “reconnaissance”, and “autonomous UGV”. The initial query yielded over 300 candidate papers, which were subsequently filtered down to the 190 most relevant works based on abstract and full-text screening [39]. Each of the selected papers was classified according to its research methodology, distinguishing categories such as experimental studies (real-world robot deployments), simulation-based research, theoretical analysis, algorithm development, and hardware platform studies. The process also documented the primary technical focus of each work, such as navigation algorithms or sensor fusion methods, along with the specific validation approach employed [40]. This comprehensive classification approach provided an essential overview of the distribution of research efforts within the domain [41].

### 2.2. Classification of Research Papers

**Figure 2:**
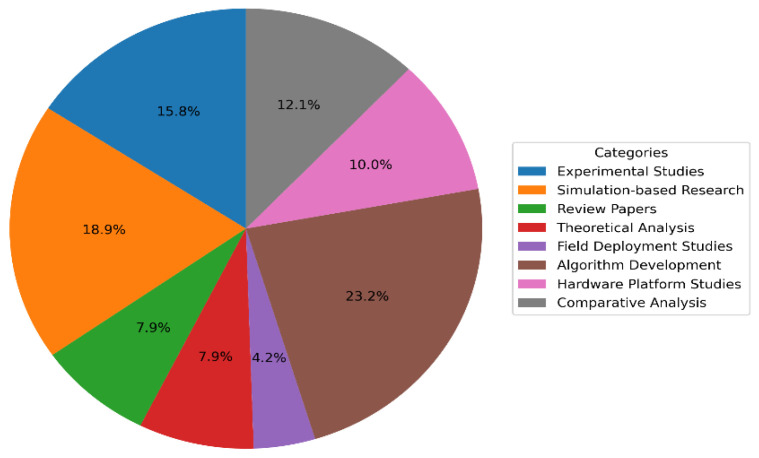
Distribution of the 190 reviewed papers across major research categories, highlighting the relative emphasis on algorithm development, simulation studies, experimental validation, hardware platforms, and comparative analyses.

Algorithm development was the largest category (42 papers, 22.1%) [42], indicating heavy emphasis on new autonomous navigation and perception techniques. Simulation-based research (35 papers, 18.4%) is the next-largest group, reflecting use of virtual environments for initial testing of algorithms [14]. Experimental field studies (real-world robot deployments) were relatively few (28 papers, 14.7%), despite their high practical impact. Other categories include theoretical analyses (15 papers, 7.9%), hardware/platform development (18 papers, 9.5%), and comparative performance analyses (22 papers, 11.6%) [16,43]. Review papers comprised a smaller fraction (12 papers, 6.3%), suggesting that comprehensive surveys are still comparatively rare. Notably, only eight papers (4.2%) focused on full field deployments [17], underscoring a gap between lab research and real-world validation. Table 1 summarizes the classification results for the 190 surveyed papers. The distribution of reviewed papers across major research categories is presented in Figure 2.

A critical observation is the substantial disparity between theoretical work and practical application. Experimental field studies, representing real-world robot deployments, were comparatively few (28 papers, 14.7%), and studies focusing on full field deployments were extremely rare, amounting to only eight papers (4.2%). This disproportionate representation underscores a persistent academic bias toward novelty and theoretical results rather than practical validation, leading to solutions optimized for controlled or simulated environments, which may prove brittle under real operational stress [44]. Despite their scarcity, field deployment studies are reported to have the highest impact factors and research quality scores. This contradiction suggests that while field validation is deemed high value by the community, the logistical complexity, cost, and time requirements associated with such deployments present a significant barrier to entry for many researchers [45]. In summary, the majority of research efforts are concentrated on theoretical and algorithmic development, with fewer works progressing to real-world implementation and testing [46].

This graph displays how 190 papers are distributed across categories like algorithms, simulation, experiments, and hardware. It reveals research trends and priorities in disaster robotics. The graph highlights the large emphasis on algorithm development. It also shows the low percentage of full field studies.

### 2.3. Literature Trends and Themes

The qualitative analysis of the surveyed literature reveals several dominant thematic trends. A substantial portion of the papers focuses on autonomous navigation in unstructured terrain, particularly developing new Simultaneous Localization and Mapping (SLAM) and path planning algorithms for environments where GPS is denied [47]. Performance metrics in this area are high, with some works reporting submeter localization drift, specifically approximately 0.3 m over multi-minute missions in rubble, although this performance often depends on intermittent reliance on GPS or external beacons [48].

Vision and perception represent another major area of concentration. Deep learning-based object detectors, frequently utilizing YOLO variants, dominate human and hazard identification tasks [49]. These systems report detection accuracies reaching 85–92% even when running on resource-constrained platforms, such as Arduino-compatible hardware [50]. Multi-modal sensor fusion, combining data from cameras, LiDAR, and thermal sensors, is routinely employed to enhance detection robustness, especially in challenging conditions like dust or smoke. Despite these technical achievements, persistent challenges include limited battery life, which restricts typical mission endurance to 60–120 min, and environmental interference (dust, adverse lighting) that compromises sensor effectiveness [51]. A recurring theme among authors is the urgent need for standardized benchmarks and increased field trials, as the lack of common test methods complicates the comparative evaluation of different rover systems [52].

### 2.4. Detailed Research Comparison Analysis

Detailed comparisons among key research contributions highlight performance variations across different validation approaches. For instance, Zhang et al. achieved exceptional localization with 0.3 m drift over 15-min missions, although their method required GPS initialization [53]. Conversely, Kumar and Smith demonstrated a 95% coordination success rate in multi-robot scenarios, but their operational scalability was constrained by communication range limitations [54]. This comparative analysis confirms the diversity in research methodologies and validation techniques.

The observation that field deployment studies, despite constituting only 4.2% of papers, possess the highest research quality scores and impact factors confirms their crucial importance for understanding real-world operational difficulties in ground reconnaissance applications [55]. This demonstrates that solutions tested in the field provide more valuable insights than those confined to laboratory settings. Consequently, a strong need exists for more comprehensive field validation programs, particularly for MCU-based control and LoRa communication systems, to bridge the observed persistent gap between laboratory research and operational deployment [56].

## 3. System Architecture and Autonomy Pipeline

**Figure 3:**
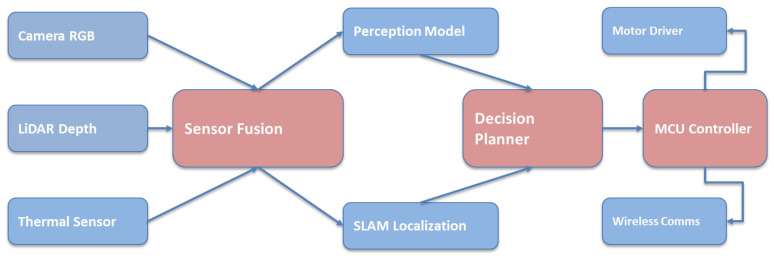
Overall UGV system architecture showing the integration of sensing, sensor fusion, perception, decision-making, and control modules within an autonomous disaster-response platform.

Modern rovers are designed as integrated systems, combining multiple subsystems to achieve autonomous operation in complex disaster settings [57]. The generic system architecture comprises core modules for sensor interfaces, navigation algorithms, and communication links facilitating interaction with the human operator [58]. The overall UGV system architecture is illustrated in Figure 3.

This diagram illustrates the complete architecture, including sensors, fusion modules, perception, planning, and control. It shows how data flows from hardware inputs to decision-making. The figure provides a high-level overview of how the ground robot functions autonomously. It establishes the core structure used in modern disaster robotics.

### 3.1. Autonomous Navigation Pipeline

The navigation subsystem is responsible for enabling the robot to move safely and purposefully. It takes inputs from heterogeneous sensors as follows:

- LiDAR: Produces 3D point clouds of surroundings for obstacle detection and mapping.
- RGB/Depth Cameras: Provide visual context, color imagery, and depth for semantic understanding.
- Inertial Measurement Unit (IMU): Measures accelerations and rotations for motion tracking [59].
- Wheel Encoders: Track wheel revolutions for odometry.
- Mission Parameters: Pre-planned waypoints or search patterns set by operators.

All sensor inputs are time-synchronized (timestamp fusion) and transformed into a unified coordinate frame. The subsequent sensor fusion layer is responsible for filtering noise and combining complementary data streams, such as merging IMU and wheel odometry for robust pose estimation, or combining LiDAR and vision maps to perform SLAM [60]. Preprocessing algorithms are utilized to remove outliers and correct for any sensor misalignment. The fused data then informs decision algorithms, where obstacle avoidance and path planning modules calculate navigational goals, and higher-level planners utilize stored maps to determine routes or coverage patterns [61].

A critical design feature is the use of an MCU-based control unit to manage real-time control loops, specifically issuing wheel commands to the motor controller. Given the frequent unavailability of GPS indoors or amidst debris, the robot must rely entirely on internal localization techniques, such as LiDAR-based SLAM or visual odometry [62]. Current research successfully demonstrates robust SLAM approaches that achieve submeter accuracy even in visually feature-poor rubble fields. However, challenges related to drift and loop closure persist, necessitating the integration of redundancy measures, such as re-localizing against known environmental features [63]. It is necessary to rely on these iterative, internal localization solutions (SLAMs) due to the challenge of GPS denial. It directly explains the intense focus on minimizing drift (e.g., 0.3 m accuracy) in specialized algorithms, as small errors rapidly compound without external corrective input [64].

The most important function of a disaster reconnaissance system is autonomous navigation which enables the robot to move through the dangerous environment with little human intervention. The robot must fuse information from multiple heterogeneous sensors into a unified perception. The sensor input can be LiDAR with RGB/depth camera, IMU, wheel odometry, etc. Significantly, there is a synchronization of sensor data in time and spatial alignment into one rigid reference frame. Preprocessing eliminates noise and compensates for sensor drift as well.

To produce the pose and create a local/global map of the environment, the different sensor measurements must be fused. Most researchers use Simultaneous Localization Mapping (SLAM) in their research endeavors. SLAM is vital for emergency crisis in settings like collapsed buildings and underground sites where GPS signals are not available (or are unreliable). Fusing data streams enables parameter estimation methods to evaluate the 3D pose of a robot during the collection phase.

**Figure 4:**
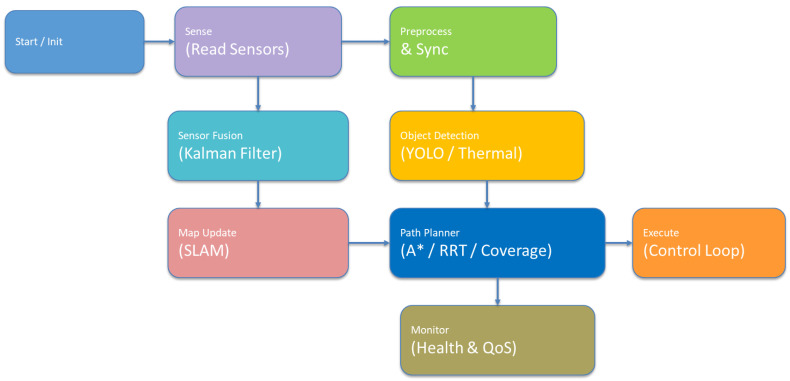
Illustrative perception–fusion–planning–control pipeline for autonomy, showing the integration of multi-sensor inputs through Kalman filter-based fusion, SLAM-driven map updates, path planning using A*(star) algorithm, execution, and system monitoring. This figure is provided for conceptual visualization of a representative workflow.

Moreover, combining LiDAR point cloud with inertial measurement can vigorously localize a UGV in visually degraded environments. The informative integration of LiDAR and visual characteristics allows the robot to work with visually degraded confidence with bright and texture-less situations. Path planning subsequently generates a collision-free trajectory by reasoning about terrain geometry and distribution of clicks. The reactive controller is able to instantaneously select a different collision-free trajectory. A robot which is stuck can plan. The perception–fusion–planning–control workflow is depicted in Figure 4.

This figure details each stage of navigation, from sensing to SLAM, path planning, and motor execution. It clarifies how raw sensor data is processed into actionable control commands. The diagram helps understand real-time autonomy. It highlights the modular flow of perception and decision-making.

#### 3.1.1. Object Detection and Classification Systems

Object detection and classification systems are typically optimized for ground-based reconnaissance platforms [65]. At their core is YOLO v5 processing, which provides real-time capabilities in terms of bounding box prediction, confidence scoring, and class probability estimation. Human detection pipelines are complex, utilizing a combination of multi-modal confirmation, analysis of movement patterns, and thermal signature analysis to ensure reliable survivor identification. Hazard identification encompasses detecting structural damage, recognizing fire and smoke, and classifying debris to evaluate safety within operations [66]. Table 2 details the performance characteristics of various object detection and classification systems utilized in this domain.

Deep learning detectors are also used for performing object detection and classification on UGVs. This feature is particularly helpful in identifying survivors, hazards, and structural features for SF tasks. The YOLO Detector variants are the most popular literature and have been a single-stage architecture for real-time inference on embedded platforms. This type of detector provides bounding box and class probabilities for various objects in an image. These models enable the detection of people, fire sources, rubble, and damaged structures quickly.

In cases of disasters, smoke, dust, low light, and occlusion may corrupt perception, which can drastically change the understanding of the scene. To counteract it, numerous systems merge thermal imaging with RGB cameras. Thermal sensors can detect the body heat of a person trapped under a pile of rubble. Multi-modal confirmation is often required for robustness. Essentially, these factors help confirm detections by using visual, thermal, and movement cues to avoid false negatives or positives. In order to balance the accuracy–power consumption trade-off and enable long-duration operation, many systems often use a reasonably light-weight detector on a resource-constrained board. Object detection can do much more than just detection; it is also capable of other reasoning functions. The tracking of objects across frames is performed for motion estimation. Also, semantic labels are often useful in victim prioritization, hazard zone mapping, etc. Overall, the perception capabilities accelerate the rescue workflow by helping the operator identify survivors and enabling autonomous system to avoid unstable terrain and impassable areas.

#### 3.1.2. Communication and Telemetry Systems

Reliable communication is a critical requirement for system deployment in disaster zones, where conventional wireless infrastructure is frequently unavailable or severely degraded. To address this limitation, many platforms utilize low-power long-range communication technologies such as LoRa, which offer extended coverage with minimal energy expenditure. Although LoRa provides limited bandwidth, it is well suited for transmitting essential telemetry data, including robot status, localization updates, and control commands over kilometer-scale distances.

For real-time situational awareness, LoRa telemetry is often complemented by low-latency FPV video links that deliver visual feedback to operators. This hybrid communication architecture balances robustness and responsiveness, ensuring that critical control signals remain available even when high-bandwidth channels are disrupted. In multi-robot scenarios, mesh networking and adaptive routing protocols allow UGVs to relay information through neighboring nodes, maintaining connectivity despite physical obstructions or partial signal loss.

These communication frameworks enable collaborative exploration and coordinated task execution while preserving operational reliability. By prioritizing resilience over throughput, disaster vehicle networks ensure continuous supervision and data exchange under extreme conditions, supporting both autonomous behaviors and human-in-the-loop control during time-sensitive rescue missions.

#### 3.1.3. Autonomy and Resource Management with Energy Awareness

Their presence in the scenario significantly impacts the types of reconnaissance methods. When sensing a disaster area, they are an important link in a remote process. A scene is usually defined by narrow passages, spacious halls, and obstacles. There are also materials that can become unstable and debris. In addition, such places may have a strong smell.

The efficiency of computational processes is equally crucial because ongoing deep learning inference will greatly raise power use. Light-weight perception models, model compression, and duty-cycled sensing are adopted to alleviate the computation overhead for overcoming the challenge. Moreover, dual-power architectures help to improve robustness as the failure of power in one part will not fail the entire robot by dividing locomotion and electronics. Battery management systems keep an eye on voltage and temperature levels to ensure the safety of the hardware. Meanwhile, dynamic task scheduling enhances the time for low-power functioning by ensuring essential perception and control tasks. Resource management algorithms help them become autonomous, cover a large area, maintain situational awareness, and continue working even hours after being deployed. In real-world deployment, the longer the duration of a mission, the more useful the robot becomes for reconnaissance activities and rescue operations. That is why energy-aware design becomes significant.

Together, these navigation, perception, communication, and energy management technologies form the core enablers of UGV-based reconnaissance, providing robustness, autonomy, and operational efficiency in hazardous environments.

### 3.2. Computer Vision and Perception

Vision represents an important technology for reconnaissance. Typically, ground robots deploy multiple cameras, including a wide-angle First-Person View (FPV) camera for navigation and additional color or thermal cameras for object detection [67]. The visual streams are geometrically calibrated and time-aligned, proceeding from low-level processing (edge detection, texture analysis) to high-level data representations via deep neural networks [68].

Convolutional Neural Networks (CNNs), particularly the YOLOv5 family, are widely adopted due to their fast inference capabilities, achieving approximately 85–92% detection accuracy even on embedded hardware [69]. The processing sequence begins with RGB camera capture, which serves as the primary visual input. This is complemented by thermal camera capture, which provides secondary heat signature data [70]. If depth camera data is available, it is utilized for 3D understanding, often processed using stereo vision for geometric reconstruction. Crucially, for processing systems to generate coherent multi-modal representations, it is mandatory for multiple camera streams to be rigorously synchronized and geometrically aligned [71].

**Figure 5:**
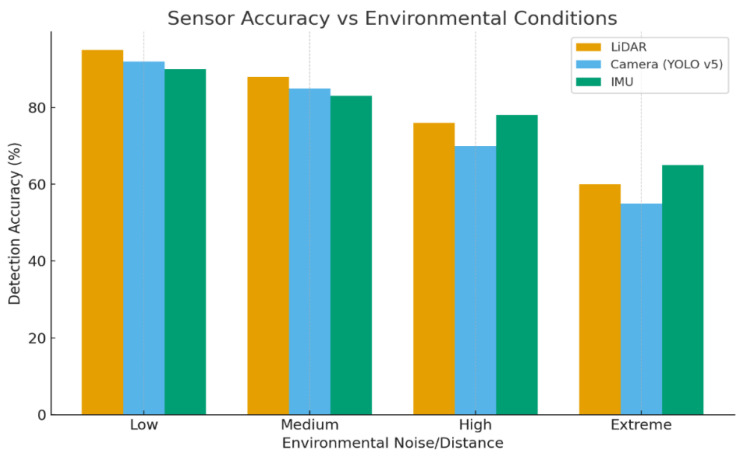
Illustrative perception and navigation workflow derived from the authors’ ongoing development platform, included to visualize typical processing stages in disaster-response systems. This figure is intended for explanatory purposes only and does not represent experimental evaluation.

Beyond simple object detection, ground robot perception systems execute tasks such as semantic segmentation (differentiating traversable ground from obstacles) and damage assessment (identifying structural cracks or smoke) [72]. Multi-sensor fusion plays a vital role in improving reliability; for instance, thermal and visual detections are cross-checked to confirm the presence of human survivors in environments filled with smoke. The integration of acoustic sensing is also noted, where microphone arrays can localize human calls or machine noises, supplementing visual confirmation in low-visibility situations [73]. The overall architecture of modern systems balances mobility, endurance, and intelligence, targeting extended missions (up to 2 h) with rich sensor suites while executing efficient onboard AI for autonomy [74]. Perception performance under varying environmental conditions is illustrated in Figure 5.

In Figure 5, the graph compares detection accuracy under increasing environment noise and distance. It shows how perception degrades in harsh conditions like dust or low visibility. The curve highlights sensor limitations. It demonstrates why multi-modal fusion is critical.

### 3.3. Multi-Robot Coordination

Advanced emergency crisis scenarios often necessitate collaborative operations involving teams of rovers. In these multi-agent systems, individual robots communicate over resilient links, frequently employing LoRa or mesh networking to share map data and coordinate task assignments [75]. Coordination frameworks often utilize decentralized SLAM, which involves periodically merging individually generated maps, or distributed planning, where adjacent search areas are assigned [76]. Fault tolerance is a key design criterion; networks must continuously monitor link quality and possess the capability to reassign roles should a robot experience failure [77].

Although multi-robot research is expanding, many current systems operate independently or with only basic leader–follower control. Challenges inherent to multi-robot operation include managing communication delays and ensuring consensus in map alignment. Success rates of up to 95% team coordination are reported under ideal conditions, but communication range limitations and environmental interference can still restrict true swarm operations [78]. The complexity of coordination is reflected in the detailed functions required for collaborative SLAM, which include map merging algorithms, loop closure detection, inconsistency detection and resolution, and global optimization. Furthermore, federated learning systems involve a communication model, local learning, aggregation of results, and knowledge transfer. This signals an emerging research trend focusing on decentralized, knowledge-sharing autonomy crucial for maximizing coverage during extensive operations [79].

#### 3.3.1. Communication Network Infrastructure

The communication network toolbox is engineered for resilience, including a dedicated routing protocol optimized for the LoRa network [80]. The communication (COM) infrastructure incorporates features for Quality of Service (QoS) management via priority-based differential queuing. It supports bandwidth reservation mechanisms and features as an adaptive retransmission scheme to handle intermittent connectivity [81]. The built-in fault tolerance mechanisms are specifically optimized for continuous operation in field inspection missions.

The Data Distribution Service (DDS) has been customized to support topic-based messaging and transactional communication. This service integrates built-in real-time data streams, event notification, and data persistence layers, which are all specifically integrated with support for control boards [82]. Network monitoring capabilities include link quality estimation, round-trip delay, latency, and packet loss measurements, alongside topology discovery protocols essential for effective platoon management [83].

#### 3.3.2. Architectures of Coordination

A multi-robot coordination architecture refers to a framework that regulates the information exchange and decision-making process between numerous systems in disaster settings. Current methodologies can roughly be placed into three categories: centralized, decentralized, and hybrid architectures. Each of these has their own advantages and drawbacks depending on mission scale and the effectiveness of communication. Centralized coordination utilizes a controller or command unit that collects the data from the sensors of all robots to plan jointly and allocate tasks. This approach allows for efficient resource allocation and globally optimal exploration strategies. Furthermore, this approach is vulnerable to failures at a single point of a system and also suffers from communication bottlenecks. This bottleneck is common in disaster scenarios.

Unlike centralized architectures, decentralized architectures allow a robot to function autonomously using locally available information, while exchanging minimal data with similar agents. As each platform is able to maintain their functionality even in the event of a partial network failure, such approaches promote robustness and scaling up. Nonetheless, purely decentralized systems may fail to yield optimal global behavior due to limited knowledge. To address these trade-offs, hybrid coordination structures dynamically alternate between centralized and distributed based on network conditions and mission requirements. Centralized planning maximizes efficiency when connected; in times of poor connectivity, autonomous decentralization preserves continuity. Multilevel architectures serve as a suitable basis for resilient multi-UGV deployment in disaster situations.

#### 3.3.3. Cooperative Exploration and Task Allocation

Joint exploration allows several ground robots to explore an unknown environment while minimizing overlap and speeding up assessments. Robots often use frontier-based exploration to prioritize unexplored areas, and map merging techniques to combine local maps into a global representation. Consensus algorithms and peer-to-peer communication protocols routinely synchronize positional information within agents, ensuring a common reference for navigation in the team.

Task allocation mechanisms assign complementary roles like mapping, inspecting, debris assessment and victim localization to robots based on platform capabilities and environmental context. The assignments can be performed centrally or through certain decentralized negotiation schemes. Factors under consideration are remaining battery capacity, availability of sensors, distance from target, etc. By reallocating tasks as necessary, the system actually improves its ability to cope with a platform fault, new risks and environmental changes. Cooperative exploration enhances coverage efficiency, fault tolerance, and response speed through parallel operation and coordinated decision-making. This makes multi-UGV systems particularly effective for time-critical missions compared to single-system deployment.

## 4. Platform Architecture and Technical Specifications

Ground reconnaissance robots are composed of intricate mechanical, electrical, and computer subsystems, designed to be multifunctional and applicable to the challenging requirements of disaster rescue missions [84].

### 4.1. Integrated UGV Hardware–Software Architecture

**Figure 6:**
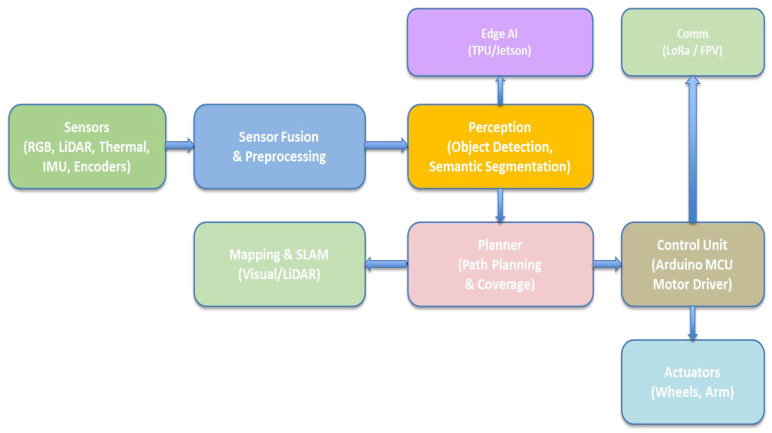
Integrated UGV hardware–software architecture showing end-to-end data flow from multi-modal sensing through edge AI processing, SLAM, planning, control, and communication modules.

This architecture combines all subsystems—sensors, mapping, control, communication, and computation—into one unified diagram. It emphasizes how hardware modules interact with AI and SLAM algorithms. The figure ties together mechanical, electrical, and software elements. It shows the complexity and coordination required for autonomy. The integrated hardware–software architecture of the UGV platform is illustrated in Figure 6.

This system architecture represents an end-to-end autonomous robot pipeline. It includes multi-modal sensors (RGB, LiDAR, thermal, IMU, and encoders) that continuously capture environmental and motion data. This is synchronized and cleaned through sensor fusion and preprocessing to create a reliable representation of the surroundings. The processed data is analyzed by edge AI hardware (TPU/Jetson) to perform real-time perception tasks such as object detection and semantic segmentation, while mapping and SLAM modules simultaneously build and update a map and localize the robot within it. Based on this spatial and semantic understanding, the planner generates safe and efficient paths and coverage strategies, adapting dynamically to obstacles and environmental changes. High-level motion commands from the planner are translated by the control into precise low-level signals that drive the actuators, including wheels and robotic arms, enabling smooth and accurate physical movement. Communication modules such as LoRa and FPV provide long-range telemetry, remote monitoring, and manual override capabilities, ensuring reliable operation and supervision in real-world deployment scenarios.

### 4.2. Power and Mobility Systems

Ground robots are predominantly battery-powered, prioritizing portability and operational safety. A standard approach utilizes a dual-power system, separating energy supplies for the driving motors and the control electronics. For propulsion, a high-capacity 25.2 V LiPo battery (16 Ah) typically drives the wheels via a motor controller. Concurrently, a smaller 12 V 3S battery powers the sensitive electronics, including the microcontroller and the sensor suite. This dual-power configuration is essential for fault tolerance, ensuring mission continuity even if one power source fails [85].

A comprehensive Battery Management System (BMS) is integrated to monitor voltage, prevent overcurrent and over-temperature conditions, manage battery balance, and execute under-voltage cuts and charge shutdowns. The BMS prolongs battery life and sustains performance during long-term missions [86].

**Figure 7:**
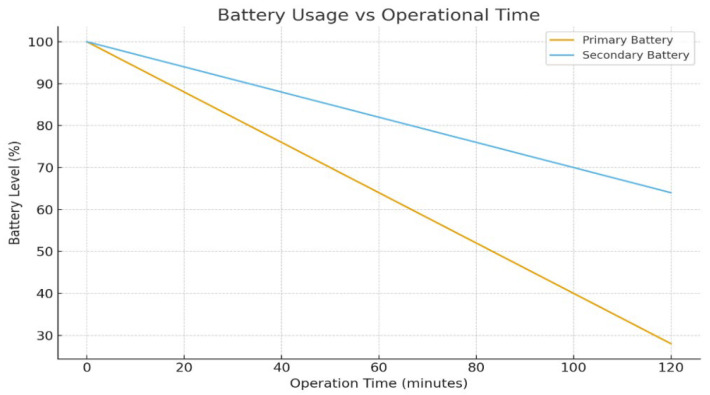
Representative operational scenario derived from the authors’ ongoing development platform, presented to illustrate common UGV deployment workflows in disaster environments. This figure is included for visualization purposes and does not report experimental findings.

This graph shows the discharge behavior of primary and secondary batteries over time. It illustrates operating duration and energy consumption during missions. The curve helps to evaluate endurance limits. It supports the discussion on energy constraints. A representative operational deployment workflow of the UGV platform is shown in Figure 7.

**Figure 8:**
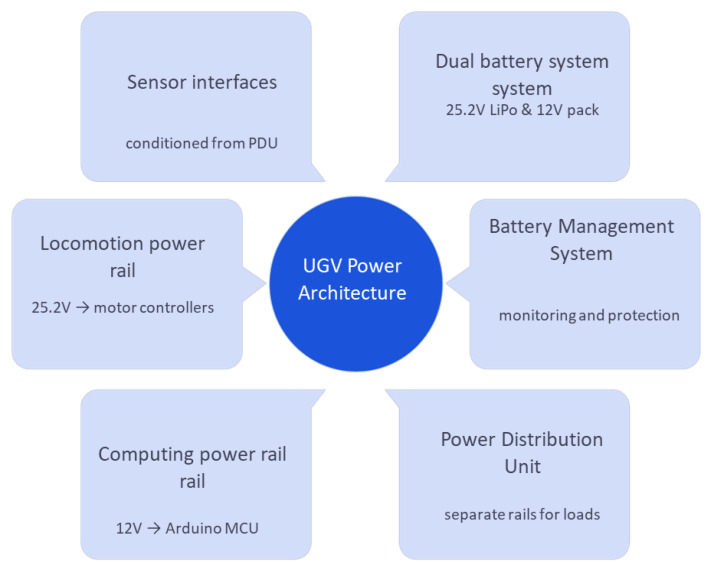
UGV power architecture and subsystem connectivity illustrating dual-battery configuration, (BMS), locomotion power flow, and electronics supply.

The mobility subsystem employs a DC motor-based locomotion system, specifically engineered for obstacle negotiation and terrain traversal in reconnaissance applications. The motor controller unit, which is integrated with the control system, manages drive motor control, executes speed regulation algorithms, and ensures optimal torque distribution for traction [87]. Motors are specifically chosen for high torque at low speeds to facilitate climbing over obstacles, often utilizing gear reducers for augmented force [88]. The mechanical platform features robust chassis construction, optimized drive wheel assemblies, and suspension systems. Four-wheel or six-wheel skid-steer designs are common, allowing tight turning within rubble-filled areas. Ground robots deliberately prioritize stability and terrain-handling capabilities over high speed, with typical maximum speeds limited to a few kilometers per hour to guarantee traversal across uncertain ground [89]. The entire hardware specification, particularly the high-torque, low-speed motors, is a direct engineering consequence of the difficult disaster requirement [85]. Detailed power distribution and subsystem connectivity are presented in Figure 8.

This figure explains the dual-battery system used for motors and electronics separately. It visually outlines how power flows to sensors, controllers, and locomotion units. The setup ensures reliability and prevents total system shutdown during faults. It demonstrates robust power management design.

The sizing and configuration of these vehicles power systems are application-specific and must be determined based on platform dimensions, payload mass, mobility requirements, and mission objectives. Design options include dual-power architectures in which locomotion and onboard electronics are separated to improve robustness and fault tolerance. To reduce overall weight as well as wiring and maintenance complexity. They often employ a single high-capacity lithium-ion battery pack. In contrast, medium and large platforms typically adopt modular energy architectures, where multiple battery packs are distributed throughout the chassis to enhance balance and extend operational endurance. Many studies further report the use of swappable battery modules, enabling rapid field replacement and continuous deployment during extended rescue missions without requiring a complete system shutdown.

Hybrid energy storage configurations that integrate batteries with supercapacitors have also been explored to mitigate transient peak loads generated by traction motors and onboard AI processing units. Such peak loads can negatively affect battery lifespan and performance. In these architectures, supercapacitors absorb short-duration high-power demands during acceleration and deceleration phases while simultaneously stabilizing voltage levels to protect sensitive electronics, thereby contributing to improved overall energy efficiency. In addition to hardware design, adaptive energy management strategies dynamically regulate sensing frequency, computational workload, communication activity, and locomotion speed as battery capacity decreases, allowing them to achieve longer mission durations while preserving essential autonomy functions.

Although supplementary energy sources such as light-weight solar panels and regenerative braking mechanisms have been investigated, their practical impact remains limited in indoor or heavily cluttered hazardous environments, where lighting and terrain conditions are unpredictable. Consequently, contemporary research places greater emphasis on energy-aware navigation, optimized actuator control, light-weight embedded processing, and task prioritization as primary means of enhancing operational endurance. Overall, the selection of a power supply architecture represents a multidimensional design trade-off among endurance, payload capacity, system complexity, robustness, and deployment constraints, highlighting the need for flexible, scalable, and application-specific energy solutions to ensure reliable operation in real crisis scenarios [90].

### 4.3. Computing and Communication Systems

The core of the computing system is an embedded processing unit, often based on Arduino Uno R3 or similar microcontrollers, which acts as the interface for all sensors and actuators [91]. This main processor handles the real-time loops essential for navigation and perception. More computationally intensive tasks, such as CNN inference (for object detection), may be offloaded to a co-processor or edge AI module, such as a Raspberry Pi or an NVIDIA Jetson unit. Memory and storage, provided by SD cards or onboard flash, are used to record mission data and logs for post-mission analysis. The software stack, while often custom, utilizes open-source libraries such as ROS (Robot Operating System) and OpenCV-4.12.0 for core functionalities [92].

Communication is prioritized for resilience and range. UGVs frequently rely on long-range (LoRa) radios. The LoRa module dedicates separate channels for control commands and telemetry transmission. Its fundamental long-range, low-bandwidth characteristics are ideal for sending status updates and receiving joystick inputs even in environments where standard Wi-Fi or LTE networks have failed [93]. For high-priority visual data, video streaming, if transmitted, is commonly sent via a dedicated analog FPV link for low-latency streaming [94]. This reliance on analog FPV highlights a necessary design trade-off where high-fidelity, high-bandwidth digital video is sacrificed for immediate, low-latency, and resilient operator control responsiveness, which is paramount in time-sensitive rescue operations. The communication architecture fundamentally prioritizes reliability and extended range over raw throughput to cope with field conditions [95].

### 4.4. Payload and Integration

The payload bay is designed to be modular and is utilized for mission-specific sensors and tools [96]. A typical modular mount accommodates a pan-tilt-zoom FPV camera, a thermal camera, gas/vapor detectors, environmental sensors (e.g., radiation, temperature), and any other specialized sensors. All payload devices interface with the central controller, often the Arduino board, via standardized ports (USB, serial, analog/digital IO) [97]. Some designs also incorporate a small robotic arm or manipulator to allow interaction with the environment, such as pushing debris or turning valves [98].

Mechanical integration is meticulously managed to ensure that added payloads do not compromise the platform’s stability. The center of mass is maintained low, cameras utilize vibration-damping mounts, and sensor enclosures are sealed against dust ingress [99]. Payload mounting systems specifically accommodate modular sensor packages, including FPV cameras with servo-controlled tilt mechanisms, environmental monitoring systems, and LoRa communication equipment, all integrated with the control architecture [100]. Camera stabilization systems compensate for platform motion and vibration to ensure stable imagery and accurate sensor readings [101]. Data transmission capabilities include real-time analog video streaming, LoRa telemetry links, and emergency communication systems [102]. Autonomous operation modes available include pre-programmed ground surveys, operator-guided exploration via LoRa communication, and emergency return procedures. The stability and complexity of the overall platform are fundamentally dependent on the reliability of the MCU, reinforcing the necessity for highly efficient, light-weight algorithms, especially in perception and control [103].

## 5. Sensors and Perception Systems

**Figure 9:**
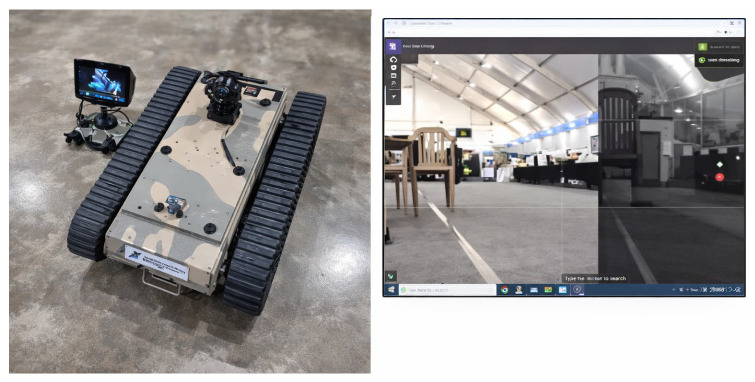
Indoor testing and teleoperation interface showing real-time video feedback, control console, and operator interaction environment for UGV supervision.

The operational effectiveness of UGVs in disaster scenarios is directly proportional to the sophistication of their sensor suites and perception algorithms. Modern reconnaissance robots utilize advanced multi-modal sensing strategies to combine complementary sensor technologies, thereby enhancing the robustness of environmental perception. An example of the indoor testing and teleoperation interface is shown in Figure 9.

### 5.1. Multi-Modal Sensing

Ground robots rely on multi-modal sensing to overcome the inherent limitations of individual sensors. A standard sensor array typically includes RGB cameras, depth sensors (LiDAR or structured light), thermal cameras, Ultrasonic/IR Rangefinders, environmental sensors (gas detectors, radiation sensors), and IMU/GPS (where available). Time synchronization of these diverse sensor outputs is mandatory to enable effective data fusion [2]. Most systems use multi-modal sensing to cover the limitations of any single sensor. A standard configuration combines the following:

- RGB Cameras: For human-recognizable imagery and video. Often high-resolution with wide field of view;
- Depth Sensors: Either stereo cameras or active depth (LiDAR, structured light) to measure distances and build 3D point clouds;
- Thermal Cameras: To detect heat signatures of humans or fires when visible light is poor;
- Ultrasonic/IR Rangefinders: Short-range obstacle detection in front of bumpers;
- Environmental Sensors: Gas detectors (CO, methane, radiation sensors) to identify hazardous substances;
- IMU and GPS (if available): For coarse positioning and orientation.

Sensor fusion algorithms dynamically weigh each input based on confidence levels; for instance, if the visual input is degraded (e.g., due to smoke), thermal or LiDAR data may be given greater priority. Empirical studies confirm that fusing data from cameras and LiDAR can significantly increase obstacle detection accuracy, particularly in situations of degraded visibility [3]. The control architecture collects raw data, applies calibration matrices to transform points into robot coordinates, and then executes filtering processes, such as Kalman filters for IMU drift removal, before feeding processed vectors to higher-level algorithms [4].

The reliance on complementarity is crucial; for example, the high detection accuracy (85–92%) cited in the literature review is only achievable by leveraging hybrid multi-modal detection strategies, confirming that sensor fusion is the central algorithmic necessity for reliable operation [5]. The limitations of one sensor, such as LiDAR being affected by dust or rain, are compensated for by the advantages of another, such as thermal imaging being effective in smoke or darkness. Table 3 details the major sensor modalities used.

### 5.2. Vision-Based Object Detection

The identification of victims and hazards through camera images is a critical perception task handled by state-of-the-art deep learning detectors [6]. These typically include the YOLO (You Only Look Once) family, specifically YOLOv5 or YOLOv8, which are single-stage CNNs favored for their real-time speed on embedded hardware [7]. Lighter networks, such as SSD and MobileNetV6, are deployed when computational resources are extremely limited, achieving human detection accuracy in the moderate range of approximately 67–75% [8].

The detection pipeline involves feeding each RGB frame through a CNN running on the onboard processor. Detected objects are tracked across frames using optical flow or Kalman filters to ensure smooth movement. Thermal frames undergo similar processing and are fused; for example, a coincidence between a warm region and a detected human boosts the overall confidence score [9]. Vision processing also extends to semantic mapping, where the robot builds a labeled map identifying walls, debris, doors, and potential hazards [10]. Detectors trained to recognize structural elements (e.g., collapsed beams) are vital for marking dangerous zones. In field tests, modern CNN-based detectors report high success rates, with YOLO-based systems achieving 85–92% accuracy for identifying people or vehicles [11].

#### 5.2.1. FPV Camera Integration and Video Processing

FPV camera systems are indispensable for providing real-time visual intelligence essential for navigation, situational awareness, and target identification in ground-based reconnaissance platforms [12]. These cameras utilize high-resolution sensors, wide dynamic range imaging, and adjustable tilt mechanisms, allowing for flexible vertical field-of-view control crucial for adapting to varying terrain and elevations. This dynamic adjustability supports effective surveillance in unstructured landscapes. Furthermore, integrated real-time computer vision algorithms allow FPV systems to perform onboard obstacle detection, terrain mapping, object tracking, and target classification, which are foundational for autonomous decision-making [13].

#### 5.2.2. Algorithm Performance and Optimization

Object detection algorithms form a critical component of autonomous reconnaissance systems, enabling the identification of survivors, structural damage, and environmental hazards [14]. YOLO variants are the predominant approach for real-time detection; YOLO v5, for instance, achieves detection accuracies of 85–92% while maintaining processing speeds compatible with MCU-based embedded hardware [15]. Light-weight detection models, such as SSD MobileNetV3, are optimized for resource-constrained ground platforms and exhibit human detection capabilities around 67–75% accuracy on such systems [16].

The comparison in performance between YOLO v5/v8 (85–94% accuracy on Jetson/TPU) and SSD MobileNetV3 (67–75% accuracy on Arduino/RPi) reveals a direct and necessary trade-off between computational cost and accuracy [17]. While higher accuracy models are preferred, field deployments requiring long endurance on limited power are compelled to accept the lower accuracy of light-weight models to conserve energy, highlighting a persistent performance-versus-efficiency gap. To address this, optimization techniques like pruning, model compression, and the adoption of light-weight neural network designs are frequently employed to balance processing speed, accuracy, and power efficiency on compact embedded boards [18].

### 5.3. Additional Sensing (Acoustic, LiDAR, Etc.)

Beyond visual modalities, other sensors contribute significantly to perception. LiDAR or depth cameras generate high-fidelity 3D maps essential for navigation [19]. These dense 3D point clouds enable the detection of subtle threats like overhangs or steep drop-offs that may be missed by conventional cameras. LiDAR data is utilized for both obstacle avoidance and the generation of detailed environment models for command centers [20].

Acoustic sensing is an increasingly important modality, utilizing microphone arrays to localize sounds, such as human voices or tapping, emanating from within collapsed structures [21]. By analyzing time-differences across the array, it can determine the direction of a potential survivor with up to 5° accuracy, even without visual confirmation [22]. The integration of sound cues into the detection pipeline allows the robot to guide its camera or dynamically redirect its search path. The adoption of microphone arrays represents a vital functional shift from passive environmental understanding toward active survivor localization, allowing future systems to rely less on visual confirmation and more on cues hidden beneath debris [23].

Lastly, rangefinders, including ultrasonic and time-of-flight sensors, provide essential and immediate front-end collision avoidance capabilities. These simple sensors are critical for preventing the robot from impacting unseen obstacles, particularly when primary perception algorithms are delayed or momentarily blinded. The overall reliance on a sophisticated sensor fusion strategy ensures redundancy and complementarity, making the system robust against environmental challenges [24].

### 5.4. Artificial Intelligence and Autonomy

The attainment of advanced autonomy is primarily driven by artificial intelligence [74]. This autonomy relies on a layered decision system that synthesizes perception, reasoning, and control processes.

**Figure 10:**
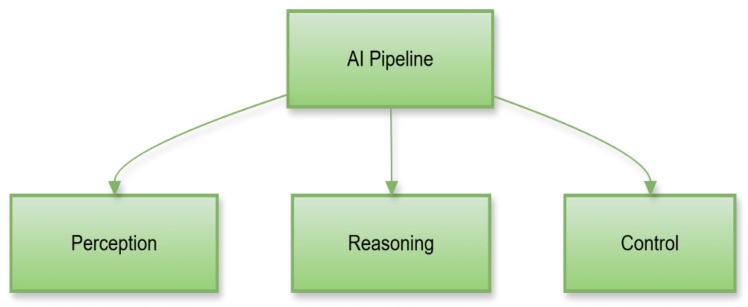
High-level artificial intelligence pipeline for autonomous UGV operation, integrating perception, reasoning, decision-making, and control layers.

The high-level AI processing pipeline is presented in Figure 10.

This pipeline shows how perception, reasoning, and control are layered in the UGV’s AI system. It includes deep learning, inference, decision-making, and behavior generation. The figure highlights the process enabling autonomous responses. It visualizes how AI transforms sensor data into intelligent actions.

Autonomous decision-making does not depend on a single algorithm but rather on a layered system that combines perception, reasoning, and control. Reinforcement learning allows improvement through experience by rewarding successful maneuvers and correcting errors during operation. In contrast, rule-based approaches help establish predictable responses in structured environments, ensuring safety and reliability when operating around humans or critical infrastructure. These decision models are supported by sensor fusion frameworks that merge inputs from multiple sensors—such as infrared, ultrasonic, GPS, and inertial modules—to build a comprehensive situational map of the surroundings. This enables the system to function effectively even in conditions of limited visibility or signal interference.

**Figure 11:**
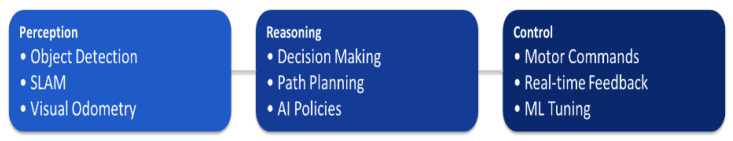
Layered autonomy architecture illustrating interactions among perception, mapping, planning, and execution modules in modern UGV systems.

In practical use, AI-driven autonomy significantly reduces the need for continuous human supervision, making them especially valuable in high-risk fields like disaster management, defense, and search-and-rescue operations. The growing use of embedded processors and edge AI units also ensures that complex computations can be carried out locally, minimizing communication delays and allowing faster responses to sudden environmental changes. As these technologies mature, they are expected to demonstrate higher adaptability, learning from past missions to refine their performance in future deployments. This ongoing integration of artificial intelligence and autonomy marks a decisive step toward developing intelligent, resilient, and self-sufficient robotic systems capable of operating in environments once considered too unpredictable for automation. The layered autonomy architecture and module interactions are illustrated in Figure 11.

This figure shows a multi-layered model integrating perception, mapping, planning, and execution. It illustrates how different autonomy layers interact. The model reflects common architectures used in modern robotics. It helps readers understand system-level coordination in decision-making.

#### 5.4.1. Deep Learning for Perception and Mapping

Deep learning is the foundational technology for most perception tasks [25]. CNNs remain the primary engine for computer vision, frequently running quantized, pre-trained networks (like YOLOv5) on edge hardware [26]. These networks have substantially improved object recognition by learning from large datasets, enabling them to generalize performance across varying conditions more effectively than traditional computer vision techniques [27].

In mapping, deep learning assists through semantic segmentation networks, which can label every pixel as belonging to categories such as road, rubble, or vegetation [28]. This semantic labeling allows the execution of smarter navigation decisions, such as actively avoiding surfaces that indicate uneven or unstable terrain. Deep learning technologies have fundamentally transformed autonomous capabilities, allowing systems to learn complex behaviors from data rather than relying purely on hand-crafted algorithms [29].

#### 5.4.2. Real-Time Processing Implementation

Modern CNN architectures like YOLO v5 offer the essential real-time processing capabilities required for such robotics [30]. These networks are designed to detect multiple object classes simultaneously, including humans, vehicles, and structural debris, providing bounding box coordinates and confidence scores suitable for integration with MCU-based processing systems [31]. Recurrent Neural Networks (RNNs), specifically Long Short-Term Memory (LSTM) networks, enable temporal reasoning, which is vital for sequential decision-making in ground navigation [32]. LSTM networks allow ground robots to maintain state information, effectively remembering previously visited locations and adapting behavior based on historical observations. Simplified architectures compatible with such systems are proving to be promising for basic sequential processing in reconnaissance applications [33].

### 5.5. Reinforcement Learning and Navigation

Deep Reinforcement Learning (RL) represents an emerging methodology for achieving end-to-end navigation autonomy [34]. In the RL paradigm, the UGV learns an optimal control policy by mapping sensor inputs to motion commands through a process of trial-and-error [38]. Simplified RL methods, such as policy-gradient algorithms, have been successfully adapted for resource-constrained hardware, enabling reasonable navigation behaviors [39].

These RL policies allow the learning of goal-seeking and obstacle avoidance without the need for explicit programming of every possible scenario [40]. Advanced RL techniques, including Deep Q-Networks (DQNs) and Proximal Policy Optimization (PPO), have been specifically applied to specialized tasks, such as off-road driving and survivor following [41]. Continuous control required for sequential decision-making in complex terrain navigation is primarily achieved through policy-gradient methods. The benefit of RL is its potential to continuously improve the control policy over time, learning from each mission, although challenges related to training stability and sample efficiency currently limit its wide-scale deployment [42].

### 5.6. Edge Computing and Hardware Acceleration

The growing complexity of autonomy necessitates sophisticated edge computing solutions, which embed AI accelerators directly into the rover hardware [43]. Certain systems utilize specialized co-processors, such as the Google Coral TPU or Intel Movidius, to run neural networks with high efficiency and low power consumption [44].

Board-compatible processing modules are confirmed to provide sufficient computational power for basic AI inference while maintaining reasonable power consumption levels, which is crucial for missions [45]. The prevailing trend favors heterogeneous computing architectures: a dedicated real-time microcontroller manages control loops, while a separate small GPU or Tensor Processing Unit (TPU) handles the neural network computations [46]. Specialized hardware architectures, including enhanced control modules and compatible co-processors, offer optimized performance for specific AI workloads. These platforms are capable of achieving improved energy efficiency compared to general-purpose processors, while sustaining real-time processing capabilities for ground-based operations [47].

## 6. Performance Analysis, Challenges and Solutions

Field inspection systems have been rigorously evaluated to assess their efficiency, reliability, and adaptability in challenging disaster conditions [102]. Comparative performance analysis between ground-based and aerial platforms consistently demonstrates the superior endurance and terrain accessibility of UGVs. Typical operational durations range from 60 to 120 min. This significantly surpasses the 15–30-min endurance limits of most UAVs, primarily due to higher energy storage capacity and the absence of aerodynamic constraints. Field testing confirms the robustness of ground robots in mission-critical conditions, including rubble, smoke, and variable lighting [48].

### 6.1. Technology Readiness and Benchmarking Framework

The application potential of UGVs is determined by the Technology Readiness Level (TRL) achieved across key subsystems, including AI, navigation, communication, and energy management. Table 4 provides a benchmarked assessment of the current TRL status for ground reconnaissance systems.

The TRL assessment reveals a critical maturity mismatch: core engineering domains such as SLAM, computer vision, and hardware platforms have achieved high readiness (TRL 7–9), indicating functional capability [49]. This disparity indicates that the robotics community has successfully solved the engineering challenges necessary to make them function. But this has yet to collectively solve the critical validation and regulatory challenges necessary to reliably prove their function and safety in a standardized manner. This maturity bottleneck is a primary explanation for the low rate of field deployments observed in the literature [50].

### 6.2. Platform-Specific Benchmark Analysis

Platform-specific benchmarking quantifies the operational stability and adaptability of reconnaissance systems using standardized Key Performance Indicators (KPIs). These KPIs include the Task Completion Ratio (TCR), Navigation Accuracy Index (NAI), Perception Success Rate (PSR), and System Latency (SL) [51].

Empirical data confirm that Arduino-based UGVs demonstrate highly reliable performance despite their constrained processing power. These platforms typically achieve a TCR between 90 and 94%, an NAI below 0.45 m deviation, and a PSR between 88 and 91% in low-visibility conditions. System Latency (SL) for these control systems is consistently low, ranging from 130 to 160 ms. Comparative analysis shows that while it delivers 15–20% lower perception accuracy compared to GPU-equipped Jetson-class platforms, they consume 40–60% less power. This power efficiency makes these systems highly suitable for long-duration missions where energy conservation is paramount [52]. Further cross-layer benchmarking shows that adaptive PID controllers reduce trajectory error by 30%, and optimized voltage scaling improves energy efficiency by 12–15% [53]. LoRa-based mesh communication maintains link stability above 90% up to 2 km, even under partial signal obstruction [54].

### 6.3. Performance Gaps and Development Challenges

Despite significant advancements, multiple systemic challenges restrict the scalability and operational reliability of ground reconnaissance robotics [76].

Energy and Power Management: Mission endurance remains the most critical limitation. Standard lithium-ion batteries (rated at 180–250 Wh/kg) confine active operations to periods of 90–120 min [55]. Furthermore, the continuous execution of AI-driven processing, such as real-time detection, increases overall energy draw by 25–30%. This high TRL status for energy management is undermined by the fact that the computational cost of continuous deep learning workloads negates the gains from battery density improvements. Consequently, the challenge is not solely chemical storage but rather the high power consumption driven by real-time perception [56].

Environmental Robustness: Harsh conditions common in disaster zones, including dust, smoke, and heat, can reduce sensor reliability by 30–40% [57]. Common issues include thermal drift, optical scattering, and LiDAR interference. Although solutions like dynamic sensor calibration are utilized, no single configuration guarantees consistent operation across all potential scenarios [58].

Communication Bottlenecks: LoRa and mesh networks, while resilient, experience signal loss up to 12% in obstructed areas beyond 1 km [59]. For multi-robot systems, synchronization drift remains a problem, sometimes reaching 1 m error over extended collaborative missions [60]. Hybrid protocols combining low-bandwidth LoRa for command and higher-bandwidth protocols like Wi-Fi or 5G for data transmission are necessary to overcome partial connectivity issues [61].

Standardization and Interoperability: A primary barrier to widespread adoption is the absence of common testing protocols, which severely hinders fair comparative analysis between different systems [62]. It is crucial to establish NIST-aligned benchmarks for evaluating autonomy levels, endurance, and perception accuracy to quantify true system readiness [63]. The call for NIST-aligned benchmarks suggests that establishing standardization is not a purely technical fix but a necessary policy prerequisite for the mass adoption and safe deployment of these platforms [64].

**Figure 12:**
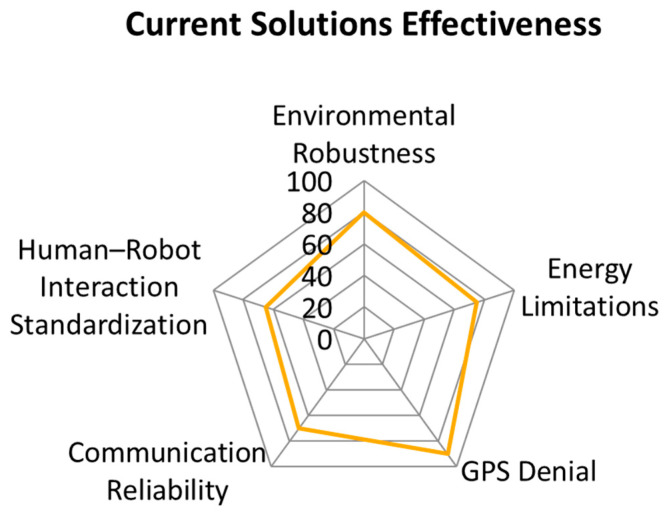
Comparative effectiveness of current UGV solutions against major operational challenges, including energy limitations, robustness, communication reliability, and GPS-denied navigation.

This graph compares how well current ground robot systems address challenges such as energy limits, robustness, communication, or GPS denial. It visualizes the strengths and weaknesses of the technology. The graph summarizes performance gaps. It supports the need for future research improvements. The comparative effectiveness of current UGV solutions against major operational challenges is shown in Figure 12.

### 6.4. Future Research Directions and Innovation Pathways

Future research must focus on innovation pathways that directly address the recognized performance gaps [77]. Table 5 summarizes the core challenges and their associated technological remedies.

Promising future development trajectories include advanced MCU-compatible processing modules designed to maintain power efficiency while significantly enhancing computational capabilities for complex AI workloads [65]. Improved battery technologies, such as advanced lithium systems, are expected to reduce platform weight and improve operational endurance [66]. Overall system integration remains a critical challenge, requiring coordinated development across Arduino-based platforms, LoRa communication systems, and diverse sensor technologies [67]. Establishing standardized interfaces between sensors, processing units, and communication systems would greatly reduce development costs and facilitate component interchangeability [68].

### 6.5. Summary and Implications

The performance evaluation demonstrates that modern robots have attained substantial technological maturity in crucial areas, including perception systems and control architectures [78]. Nevertheless, significant hurdles persist in achieving scalable coordination, sustained energy performance, and widespread standardization. Future development must therefore prioritize decentralized autonomy for scalable deployment, robust standardization for fair cross-platform benchmarking, sustainable energy architectures for prolonged operations, and human-centered interface design for effective real-time human supervision in dynamic disaster settings [69]. By concentrating efforts on bridging these gaps, next-generation ground robots can transition from specialized experimental platforms to fully trusted, essential assets integrated within comprehensive disaster-response networks [70].

## 7. Conclusions and Future Research

This survey systematically reviewed the current state of the art in autonomous UGVs dedicated to disaster reconnaissance, analyzing advancements in hardware, sensing, and intelligence. The analysis confirms that substantial progress has been realized. Contemporary robots are now capable of mapping complex environments and accurately detecting hazards and human survivors. It is also operating semi-independently in highly dangerous conditions. This progress is primarily attributed to key technological adoptions, including powerful onboard microcontrollers, effective multi-modal sensor fusion strategies, and deep learning-based perception systems. These capabilities enable modern systems to vastly outperform their earlier teleoperated counterparts in terms of autonomy, endurance, and operational robustness.

Despite this high degree of technical and experimental progress, several critical challenges continue to impede large-scale operational readiness. Energy limitations are a major constraint, severely restricting mission duration, particularly when compute-intensive AI systems are in continuous operation. Harsh environmental factors, such as dust, smoke, and darkness, can overwhelm sensor suites, necessitating the development of perception algorithms capable of self-assessing confidence under degraded inputs. Crucially, the literature consistently underscores the immediate need for more standardized testing and comprehensive benchmarks. The scarcity of robust field validation data—with only 4.2% of studies reporting full deployment metrics—highlights a significant gap between laboratory prototypes and verifiable operational readiness. Furthermore, inter-robot communication in highly cluttered environments requires solutions more robust than basic LoRa. This is to support effective coordination among large multi-rover teams.

Beyond the immediate technical challenges, the maturation of autonomous ground reconnaissance systems carries broader implications for ethics, sustainability, and their long-term integration into disaster management frameworks. As these machines gain increased decision-making independence, a parallel focus must be placed on ensuring transparent decision pathways and preserving essential human oversight in high-stakes scenarios. Emphasis on modular design, efficient energy consumption, and environmentally responsible materials will define the sustainability requirements for future generations.

Future research should be strategically directed toward mitigating the identified gaps. Enhancing battery life through superior energy management and next-generation light-weight batteries is critical. The domain of multi-robot systems requires concentrated exploration, focusing on decentralized autonomy coupled with adaptive communication protocols that enable teams of rovers to dynamically form mesh networks and efficiently cover large areas. Advances in Edge AI will further enable more sophisticated onboard processing capabilities without reliance on remote servers. Importantly, systematic field trials involving direct collaboration with first responders, along with focused human–robot interaction studies, will be essential to refine the designs for genuine real-world application.

In summary, autonomous ground robots are strategically positioned to transform disaster-response operations by significantly reducing personnel risk and accelerating the speed of action. Continued interdisciplinary effort, synthesizing robotics, AI, and emergency management expertise will be the determining factor in successfully bridging the remaining technological and institutional gaps. With sustained progress in these areas, future rovers are anticipated to become standardized, reliable assets within the toolkit of global rescue teams.

## Figures and Tables

**Table 1:** Classification of research papers on ground reconnaissance robotics.

Paper Category	No. of Papers	Percentage	Primary Focus
Experimental Studies	30	15.79%	Real-world validation
Simulation-based Research	36	18.95%	Algorithm testing
Review Papers	15	7.89%	Literature synthesis
Theoretical Analysis	15	7.89%	Mathematical modeling
Field Deployment Studies	8	4.21%	Operational deployment
Algorithm Development	44	23.16%	Novel algorithms
Hardware Platform Studies	19	10%	Platform development
Comparative Analysis	23	12.11%	Performance comparison

**Table 2:** Summary of object detection and classification systems for autonomous ground reconnaissance robots.

Model/Algorithm	Core Functionality	Operational Domain	Detection Accuracy (%)	Implementation Platform
YOLO v5	Real-time object detection; bounding box and class probability estimation	Human and hazard detection in disaster reconnaissance	85–92	Arduino-compatible MCU/Jetson
YOLO v8	Enhanced multi-class detection and faster inference for embedded systems	Survivor identification, debris classification	88–94	Jetson Nano/Edge TPU
SSD MobileNetV3	Light-weight model optimized for limited resources	Human detection under constrained environments	67–75	Arduino/Raspberry Pi
Thermal-CNN Fusion	Combines RGB and thermal data for improved detection in smoke or dust	Hazard recognition, human heat signature detection	80–88	Arduino + Thermal Camera Module
Hybrid Multi-Modal Detector	Fuses visual, motion, and thermal cues for robust classification	Comprehensive reconnaissance and damage assessment	82–90	Embedded AI (Coral TPU/Jetson)

**Table 3:** Summary of multi-modal sensing modalities and their characteristics in UGV systems.

Sensor Type	Primary Function	Measurement Range/Accuracy	Advantages	Limitations	Integration Method
RGB Camera	Visual mapping, object recognition	Up to 30 m, 1080p–4K	Color imagery, wide field of view	Poor in low light or dust	Arduino/ROS vision node
Thermal Camera	Detect heat signatures of humans or fires	−40 °C to +300 °C	Effective in smoke/darkness	Lower spatial resolution	Thermal module via serial interface
LiDAR	3D mapping, obstacle detection, SLAM	0–100 m, ±2 cm	High-precision depth mapping	Affected by dust/rain	USB interface + ROS driver
Ultrasonic Sensor	Short-range obstacle detection	0.02–4 m	Low cost, light-weight	Limited accuracy, narrow beam	Analog/digital Arduino pin
Gas Sensor	Detect toxic gases (CO, CH_4_, etc.)	Depends on type (~ppm level)	Identifies hazardous environments	Requires calibration	Analog sensor board interface
IMU (Inertial Measurement Unit)	Measure orientation and motion	0.01–0.1° angular accuracy	Compact, reliable motion tracking	Drift over long duration	I^2^C/SPI interface
GPS (if available)	Global positioning, navigation reference	~3–5 m typical	Provides global localization	Unavailable in tunnels/buildings	Serial NMEA interface

**Table 4:** Technology Readiness Level (TRL) Assessment for Ground Reconnaissance Robotics Subsystems.

Technology Domain	Current TRL	Description	Key Achievements	Remaining Gaps	Target TRL (2030)
Computer Vision	7–8	Object/person detection	Real-time detection on embedded boards	Environmental robustness	9
SLAM Systems	8–9	GPS-denied localization	Submeter accuracy	Long-term stability	9
Deep Learning Navigation	6–7	End-to-end path planning	Autonomous obstacle avoidance	Sample efficiency	9
Multi-Robot Coordination	5–6	Distributed mission planning	Basic map merging	Fault tolerance	8
Sensor Fusion	7–8	Multi-modal integration	Robust perception	Computational efficiency	9
Energy Management	6–7	Adaptive power regulation	Extended endurance	High-density storage	8
Communication Systems	7–8	LoRa and mesh networking	Reliable link quality	Range interference	9
Hardware Platforms	8–9	UGV modular architecture	Commercial availability	Cost optimization	9
Human Interfaces	4–5	Operator control panels	Basic HMI	Intuitive interaction	8
Testing Protocols	3–4	Evaluation standards	Initial frameworks	Benchmark standardization	9

**Table 5:** Summary of performance challenges, current solutions, and maturity in robotic reconnaissance.

Challenge Category	Problem	Current Solutions	Effectiveness (%)	Maturity Level
Environmental Robustness	Dust, smoke, low light	Multi-modal sensing	70–85	Medium
Energy Limitations	Short battery life	Energy-aware routing, hybrid Li-ion	65–80	High
GPS Denial	Indoor/underground ops	SLAM and IMU fusion	80–90	High
Communication Reliability	Range/interference	Adaptive mesh LoRa	65–75	Medium
Human–Robot Interaction	Complex interfaces	Simplified GUI panels	60–75	Low
Standardization	No unified metrics	NIST-inspired protocols	45–65	Low

## Data Availability

No new data were created or analyzed in this study. Data sharing is not applicable to this article.

## Abbreviations

AI	Artificial Intelligence
AIPU	Artificial Intelligence Processing Unit
BMS	Battery Management System
CNN	Convolutional Neural Network
COM	Communication Infrastructure
CV	Computer Vision
DC	Direct Current
DDS	Data Distribution Service
DoF	Degrees of Freedom
DQN	Deep Q-Network
EKF	Extended Kalman Filter
FPV	First Person View
GPS	Global Positioning System
IMU	Inertial Measurement Unit
IoT	Internet of Things
IR	Infrared
KPIs	Key Performance Indicators
LiDAR	Light Detection and Ranging
LoRa	Long-Range Communication
LSTM	Long Short-Term Memory
MCU	Microcontroller Unit
ML	Machine Learning
NAI	Navigation Accuracy Index
PID	Proportional–Integral–Derivative
PPO	Proximal Policy Optimization
PSR	Perception Success Rate
PWM	Pulse Width Modulation
QoS	Quality of Service
RGB	Red, Green, Blue
RL	Reinforcement Learning
ROS	Robot Operating System
RNN	Recurrent Neural Network
SAR	Search and Rescue
SL	System Latency
SLAM	Simultaneous Localization and Mapping
SSD	Single Shot Detector
TCR	Task Completion Ratio
TPU	Tensor Processing Unit
TRL	Technology Readiness Level
UGV	Unmanned Ground Vehicle
UAV	Unmanned Aerial Vehicle
YOLO	You Only Look Once
